# Probing the compartmentalization of HIV-1 in the central nervous system through its neutralization properties

**DOI:** 10.1371/journal.pone.0181680

**Published:** 2017-08-25

**Authors:** Karl Stefic, Antoine Chaillon, Mélanie Bouvin-Pley, Alain Moreau, Martine Braibant, Frédéric Bastides, Guillaume Gras, Louis Bernard, Francis Barin

**Affiliations:** 1 Université François Rabelais, Inserm U966, Tours, France; 2 Laboratoire de Bactériologie-Virologie, CHU Bretonneau, Tours, France; 3 Deparment of Medicine, University of California San Diego, La Jolla, California, United States of America; 4 Médecine Interne et Maladies Infectieuses, CHU Bretonneau, Tours, France; Northwestern University Feinberg School of Medicine, UNITED STATES

## Abstract

Compartmentalization of HIV-1 has been observed in the cerebrospinal fluid (CSF) of patients at different clinical stages. Considering the low permeability of the blood-brain barrier, we wondered if a reduced selective pressure by neutralizing antibodies (NAb) in the central nervous system (CNS) could favor the evolution of NAb-sensitive viruses in this compartment. Single genome amplification (SGA) was used to sequence full-length HIV-1 envelope variants (453 sequences) from paired CSF and blood plasma samples in 9 subjects infected by HIV variants of various clades and suffering from diverse neurologic disorders. Dynamics of viral evolution were evaluated with a bayesian coalescent approach for individuals with longitudinal samples. Pseudotyped viruses expressing envelope glycoproteins variants representative of the quasi-species present in each compartment were generated, and their sensitivity to autologous neutralization, broadly neutralizing antibodies (bNAbs) and entry inhibitors was assessed. Significant compartmentalization of HIV populations between blood and CSF were detected in 5 out of 9 subjects. Some of the previously described genetic determinants for compartmentalization in the CNS were observed regardless of the HIV-1 clade. There was no difference of sensitivity to autologous neutralization between blood- and CSF-variants, even for subjects with compartmentalization, suggesting that selective pressure by autologous NAb is not the main driver of HIV evolution in the CNS. However, we observed major differences of sensitivity to sCD4 or to at least one bNAb targeting either the N160-V1V2 site, the N332-V3 site or the CD4bs, between blood- and CSF-variants in all cases. In particular, HIV-1 variants present in the CSF were more resistant to bNAbs than their blood counterpart in some cases. Considering the possible migration from CSF to blood, the CNS could be a reservoir of bNAb resistant viruses, an observation that should be considered for immunotherapeutic approaches.

## Introduction

HIV-1 replication in the central nervous system (CNS) occurs early after infection [1–3] and is maintained throughout the course of the disease. It is responsible of a global neurocognitive burden that can evolve toward the fatal HIV-associated dementia (HAD) in the absence of treatment [4]. Since the advent of highly active antiretroviral therapy (HAART), HAD is rarely observed but milder forms are frequent, such as asymptomatic neurocognitive impairment (ANI) and mild neurocognitive disorders (MND). Thus, HIV-associated neurocognitive disorders (HAND) might affect as much as half of HIV infected individuals on potent HAART [5,6]. Furthermore the CNS constitutes a viral compartment that not only participates to the inflammation causing the neurologic decline (reviewed in [7–9], but is a location where viruses with specific properties such as resistance to antiviral drugs can be selected [10,11]. Therefore, improving our knowledge about the viruses infecting the CNS, their evolution and their potential role in the lifelong systemic infection is important. Distinct evolutionary patterns of viral populations in the brain and the cerebrospinal fluid (CSF) have been detected in a subset of HIV infected individuals, depending on the stage of the disease, the presence of symptoms and the methodology used [11–20]. The compartmentalization of HIV-1 in the CNS has been reported frequently in association with severe neurocognitive stages, particularly in necropsies [13,15–18]. The study of CSF from patients with milder forms of impairment revealed that viral compartmentalization was observed in up to half of patients but its frequency did not correlate with the severity of the symptoms [19–24].

While independent evolution of HIV-1 in the CNS was evidenced by genomic analysis, the replication in this specific environment relies on phenotypic properties that are not fully understood. Understanding the properties that distinguish the compartmentalized viral population could help to gain insight into this particular reservoir and the neuropathogenesis. CNS compartmentalization has been most studied through the prism of *env* gene due to the role of the Envelope (Env) glycoproteins in the cell tropism (reviewed in 11,15–16]. It has been shown that viral strains isolated from the brain or compartmentalized viral populations from the CSF tend to harbor macrophage tropism, this phenotype being associated with the capacity to infect cells with low CD4 expression at their surface [19,20,24]. In addition, the Env glycoproteins are the main targets of the humoral response. Selective pressure by neutralizing antibodies (NAb) is a major driving factor of the evolution of circulating viral population in the blood. Escape variants resistant to autologous NAb are continuously selected and identified in the bloodstream [25,26]. Some authors have suggested that the reduced selective pressure in the CNS, an immune privileged compartment, could explain some of the differences observed in the genetic sequences between CSF and blood [14,21]. Pillai et al. found a near complete lack of neutralization activity in CSF, which can be explained by the 100- to 1000-fold lower concentration of immunoglobulins in the CSF [25]. Therefore, one could suggest that, in contrast to the continuous escape observed in the blood, a reduced selective pressure by NAbs in the CNS compartment would result in a viral population more sensitive to autologous neutralization compared to its blood counterpart. In order to evaluate this hypothesis, we analyzed the genetic and neutralization properties of the viral populations present in paired CSF and plasma samples from individuals with neurocognitive disorders. Our data suggest that the selective pressure by autologous NAbs does not seem to play a major role in HIV-1 compartmentalization to CNS. In addition, using a panel of broadly neutralizing antibodies (bNAb) to analyze the phenotypic properties of the viral populations in each compartment, we observed that HIV-1 variants present in the CSF were more resistant to bNAbs than their blood counterpart in some cases. As a consequence, the CNS might serve as a reservoir for variants which would be resistant to forthcoming bNAb immunotherapies.

## Results

### Study population

Nine patients with paired CSF and plasma samples collected at onset of neurologic symptoms were included. At that time, all of them were off antiretroviral therapy (ART). Longitudinal blood plasma samples were available for four patients, but no additional sequential CSF sample was available for any of them. Characteristics of the participants and the samples are described in Table 1 and S1 Table.

**Table 1:** Clinical and virological characteristics of the study individuals and compartmentalization data.

Subject id^a^	Sample date (Mon/Yr)	Delay from CSF sampling^b^	Origin^c^	CD4^d^	VL^e^	number of SGS^f^	Analysis of compartmentalization^g^			CSF state^h^	Clade^i^
							Fst	Snn	SM		
KU	Jul-11	-288	BP		5.36	25	< 0.01	< 0.01	< 0.01	Cp	CRF02_AG
	May-12	0	BP	23	5.77	10					
	May-12	0	CSF		4.48	19					
	Jun-13	391	BP		6.31	28					
GK	Nov-05	-1978	BP		4.5	22	< 0.01	< 0.01	< 0.01	Cp	H
	Apr-11	0	BP	283	5.05	27					
	Apr-11	0	CSF		6.52	18					
	Apr-12	367	BP		5	21					
RO	Dec-10	-677	BP	48	5.99	16	< 0.01	< 0.01	< 0.01	Cp	C
	Oct-12	-1	BP		5.82	19					
	Oct-12	0	CSF		5.84	10					
KP	Oct-11	-1147	BP	222	6.56	11	< 0.01	< 0.01	< 0.01	Cp	A1H
	Dec-14	0	CSF		3.66	14					
	Dec-14	1	BP		5.79	9					
MG	Apr-08	-2	BP	-	4.77	21	< 0.01	< 0.01	< 0.01	Cp	B
	Apr-08	0	CSF		4.92	18					
BA	Mar-14	0	CSF	135	4.12	17	0.43	0.21	0.30	Eq	C
	Mar-14	3	BP		4.52	19					
BL	Apr-14	0	BP	222	5.75	18	0.21	0.08	0.06	Eq	A1
	Apr-14	0	CSF		5.12	21					
FU	Nov-13	-2	BP	55	5.87	28	0.87	0.95	1	Eq	B
	Nov-13	0	CSF		4.13	24					
SE	Apr-09	0	CSF	319	5.98	22	0.28	0.29	0.49	Eq	CRF01_AE
	Apr-09	1	BP		5.11	16					

^a^ Patients were given anonymized codes.

^b^ delay of longitudinal samples before or after the CSF sample is shown in days.

^c^ CSF = cerebrospinal fluid; BP = blood plasma

^d^ CD4 = blood CD4+ T cell count, at time of lumbar puncture, in cells/mm^3^.

^e^ VL = viral load; HIV RNA in log_10_ copies/mL.

^f^ number of single genome sequencing (SGS) amplicons.

^g^ Three methods were used to detect genetic compartmentalization between viral populations in the blood plasma and CSF: Wright’s measure of population subdivision (F_st_), the Nearest-neighbor statistic (S_nn_) and the Slatkin-Maddison test (SM); P values < 0.01 account for significant genetic compartmentalization.

^h^ CSF and blood viral populations were defined compartmentalized (Cp) based on 1) topology of the phylogenetic trees and, 2) three concordant significant compartmentalization tests (*P* < 0.01), or otherwise equilibrated (Eq).

^i^ Subtype was identified by several methods (REGA Subtying tool [27], Comet [28], HIV BLAST [29] and phylogeny using Maximum Likelihood). Subtyping was concordant for all the cases except for patient KU whose strain was classified CRF02_AG by the first three methods but other approaches suggested a more complex unique recombinant form (Recombinant Identification Program [30] and phylogeny using Maximum Likelihood).

Blood plasma/CSF pairs and longitudinal plasma samples were analyzed by single genome amplification followed by sequencing of the *env* gene. A total of 453 single genome sequences were obtained with a mean of 19 sequences (9–28) per sample. Phylogenetic analysis demonstrated the absence of cross-contamination between samples from different individuals and showed the broad diversity of the strains which were related to seven different clades (S1 Fig and Table 1).

### Compartmentalization of CSF viral population was observed in half of the subjects

**Fig 1:**
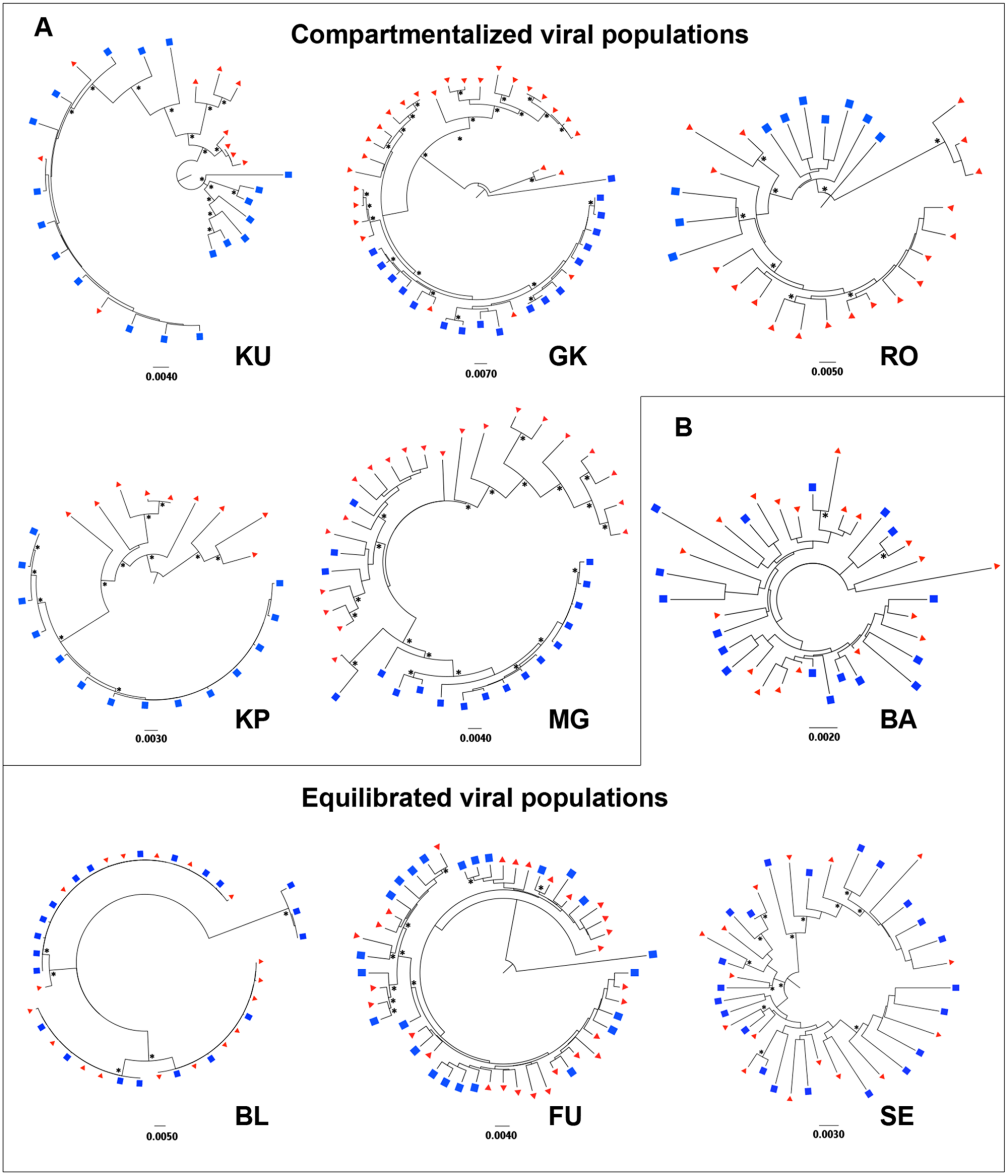
Phylogenetic relationships of paired CSF and blood plasma viral sequences. Neighbor-joining phylogenetic trees representing (A) compartmentalized and (B) equilibrated viral populations. *Env* sequences from the CSF (blue squares) and blood plasma (red triangles) are shown. Bootstrap values > 0.7 are mentioned (*) next to each node. Genetic distance is indicated with a scale bar (number of nucleotide substitutions per site).

Compartmentalization was studied for 330 sequences obtained from the contemporaneous plasma/CSF pairs of the 9 patients. It was assessed based on interpretation of neighbor-joining trees and statistical evidence using three methods [31,32]: the tree-based Slatkin-Maddison test (SM) [33] and two distance-based methods, the Wright’s measure of population subdivision (F_st_) [34] and the Nearest-neighbor statistic (S_nn_) [35]. CSF viral population was defined compartmentalized if all three tests reached significance (*P* values < 0.01) and a distinct monophyletic CSF subpopulation with boostrap value ≥ 0.70 was observed [19,20]. CSF viral population was otherwise defined as equilibrated. Five of nine patients (55%) exhibited evidence of compartmentalization with the presence of an independently evolving viral population within the CNS (Fig 1, Table 1).

**Fig 2:**
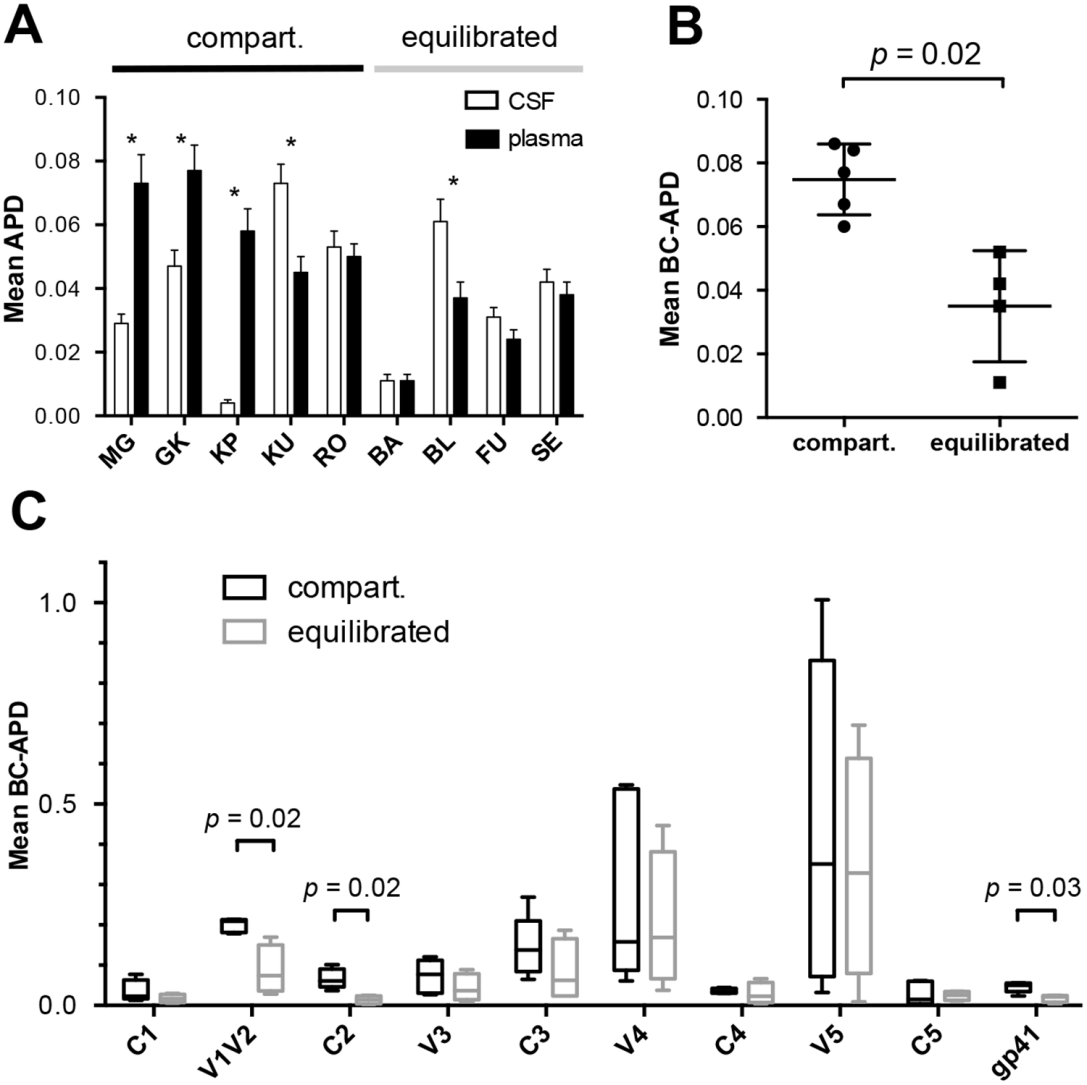
Amino acid diversity of HIV-1 *env* among contemporaneous CSF and plasma viral populations. (A) Box plot representing, for each patient, amino acid mean average pairwise distance (APD) between SGS sequences in paired CSF (white) and plasma (black) samples. Patients are grouped according to evidence of compartmentalization in CSF (* = significant difference between CSF and plasma population diversity by t-test, *p* < 0.001). (B) Mean APD between compartments (BC-APD, CSF vs. plasma) is shown for all patients. BC-APD was significantly greater for subject with compartmentalized viral populations (Mann-Whitney test, *p* = 0.02). (C) BC-APD was calculated across all regions of HIV-1 *env* for subjects with (black box plot) or without (grey box plot) evidence of CSF compartmentalization. The Wilcoxon matched-pairs ranked test and the Mann-Whitney test were used to determine *p*-values for paired observations (CSF vs. plasma) and observations between viral population structures (compartmentalized vs. equilibrated), respectively. Non-significant *p*-values are not represented. Compart.: evidence of CSF viral compartmentalization.

For each individual, we analyzed the amino acid diversity of the envelope glycoprotein (Env) sequences by determining the mean average pairwise distance (APD) within each compartment. We also analyzed the mean average pairwise distance between compartments (BC-APD). For three of five individuals with compartmentalized viral populations (MG, GK, KP), the mean APD was significant lower in CSF compared to blood plasma (t-test, *p* < 0.001) (Fig 2A). In contrast, there was no difference in APD in paired samples from three of four individuals without compartmentalized viral populations. Globally, the mean diversity between CSF and plasma variants (BC-APD) was significantly greater for individuals with evidence of compartmentalization *vs* those without compartmentalization (7.70% *vs* 3.85%, *p* = 0.02), in agreement with the tree topologies (Fig 2B). Region specific analysis revealed that the genetic distance between blood plasma and CSF sequences was significantly increased in V1V2, C2 and gp41 for individuals with compartmentalization (20.9% *vs* 7.45%, *p* = 0.02, 6.1% *vs* 1.3%, *p* = 0.02 and 5% *vs* 1.9%, *p* = 0.03, respectively) (Fig 2C).

### Longitudinal analysis revealed different evolution patterns of viral populations

**Fig 3:**
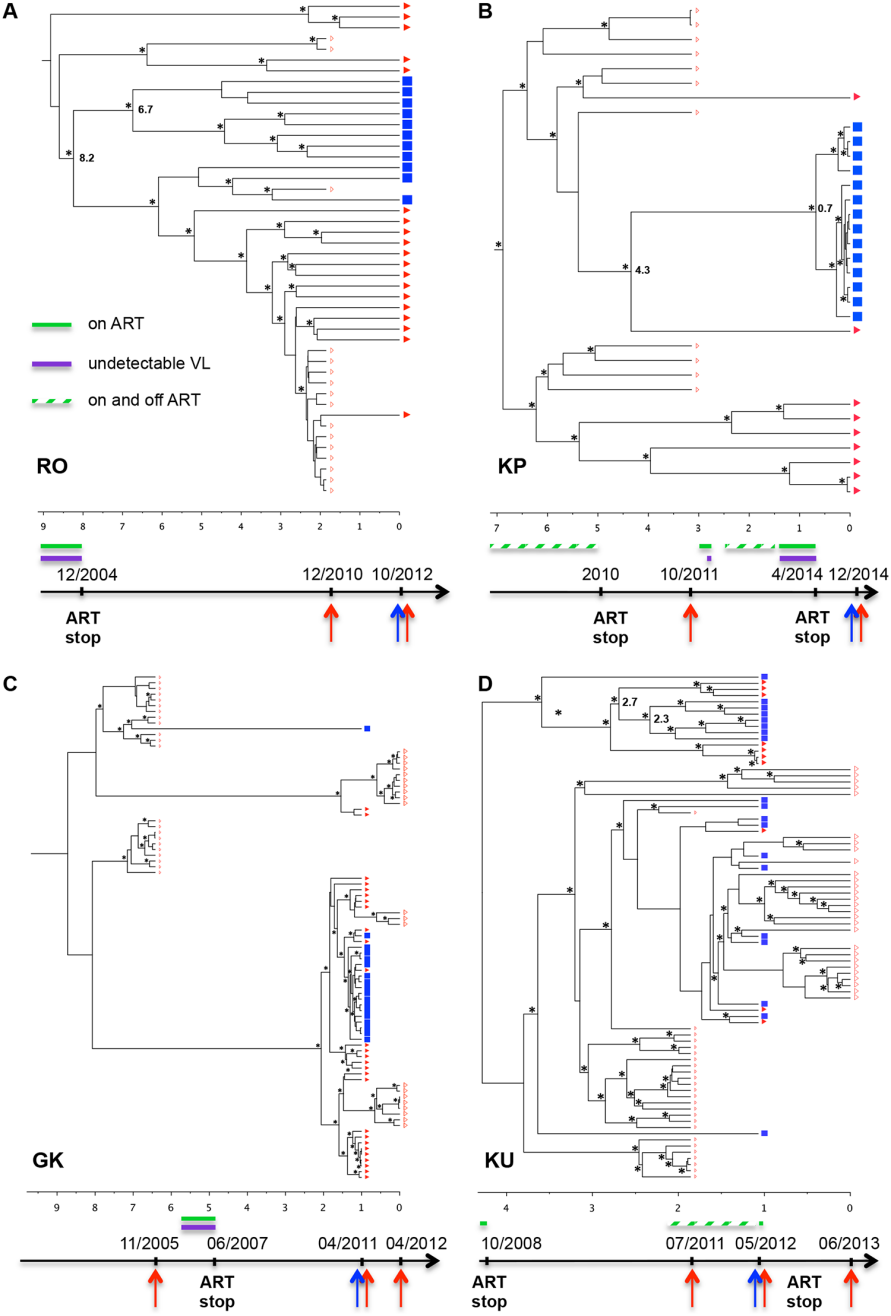
Time scaled Bayesian evolution trees for subjects RO (A), KP (B), GK (C) and KU (D). Blue squares and red triangles (full symbols at time of CSF collection, empty symbols for the other time points) represent CSF and blood plasma derived variants, respectively. Asterisks indicate posterior probability > 0.7 and timescale is in years from the last time point, e.g. the latest sample. A schematic time scale indicating the sampling dates of CSF and plasma (blue and red arrows, respectively), as well as periods of antiretroviral treatment (ART) in green and successful therapy with undetectable viral loads in purple, is shown at the bottom of each Fig. Hatched bars correspond to periods with intermittent treatment and/or poor adherence.

In order to provide insights into viral population dynamics in these patients developing neurological disorders, we performed a Bayesian phylogenetic analysis for the four subjects with longitudinal plasma samples. In three out four patients—KP, GK and KU—the viral populations were clearly divided into two major evolutionary lineages and the CSF compartmentalized populations were detected mainly in one of them, suggesting a selective advantage of one particular lineage to establish infection in the CNS (Fig 3A–3C). Time to the Most Recent Common Ancestor (TMRCA) was estimated for the viral population. However, since antiretroviral treatment (ART) could have influenced the evolutionary rate and the population dynamics, we interpreted the TMRCAs in light of the treatment history.

The longer period of divergence between plasma and CSF compartmentalized populations was observed for subject RO, with an estimated duration of 8.2 years before the time of the lumbar puncture (Fig 3A). This estimation was in agreement with the date of treatment interruption in 2004. Thus the data suggest that CNS was seeded early after viral rebound by a particular variant which underwent an extensive diversification over this period. Subject KP had a poor adherence to treatment and therefore persistent replication was observed over the years spanning the sampling dates, except for a short period during pregnancy from September 2013 to April 2014 (Fig 3B). Based on the estimated TMRCA between the CSF and plasma populations, infection of the CNS by the seeding variant could have occurred 4.3 years before onset of the neurologic symptoms. However, the estimated TMRCA of the CSF compartmentalized variants was only 0.7 years, corresponding to the treatment interruption at the end of pregnancy. A broad diversification of this lineage was observed since that date. Subject GK was on ART only during pregnancy in 2007. In contrast to cases RO and KP, it was estimated that the lineage giving rise to the CSF compartmentalized variant had diverged from the closest blood lineage less than one year before collection of the CSF sample (Fig 3C). Thus, infection of the CNS by this lineage could have been more recent. The treatment history of subject KU was more complex, with discontinuous observance and follow-up (Fig 3D). Therefore the estimated TMRCA of the compartmentalized CSF variants must be interpreted with caution.

Overall, the data suggest different patterns of evolution of CSF- and blood- viral populations. Compartmentalized variants of the CSF could have originated from variants seeding CNS years ago (up to 8 years) and could have evolved independently since then. Otherwise they could have emerged and diversified a shorter time before neurocognitive impairment.

### Compartmentalization is not associated with sensitivity to autologous neutralization

**Fig 4:**
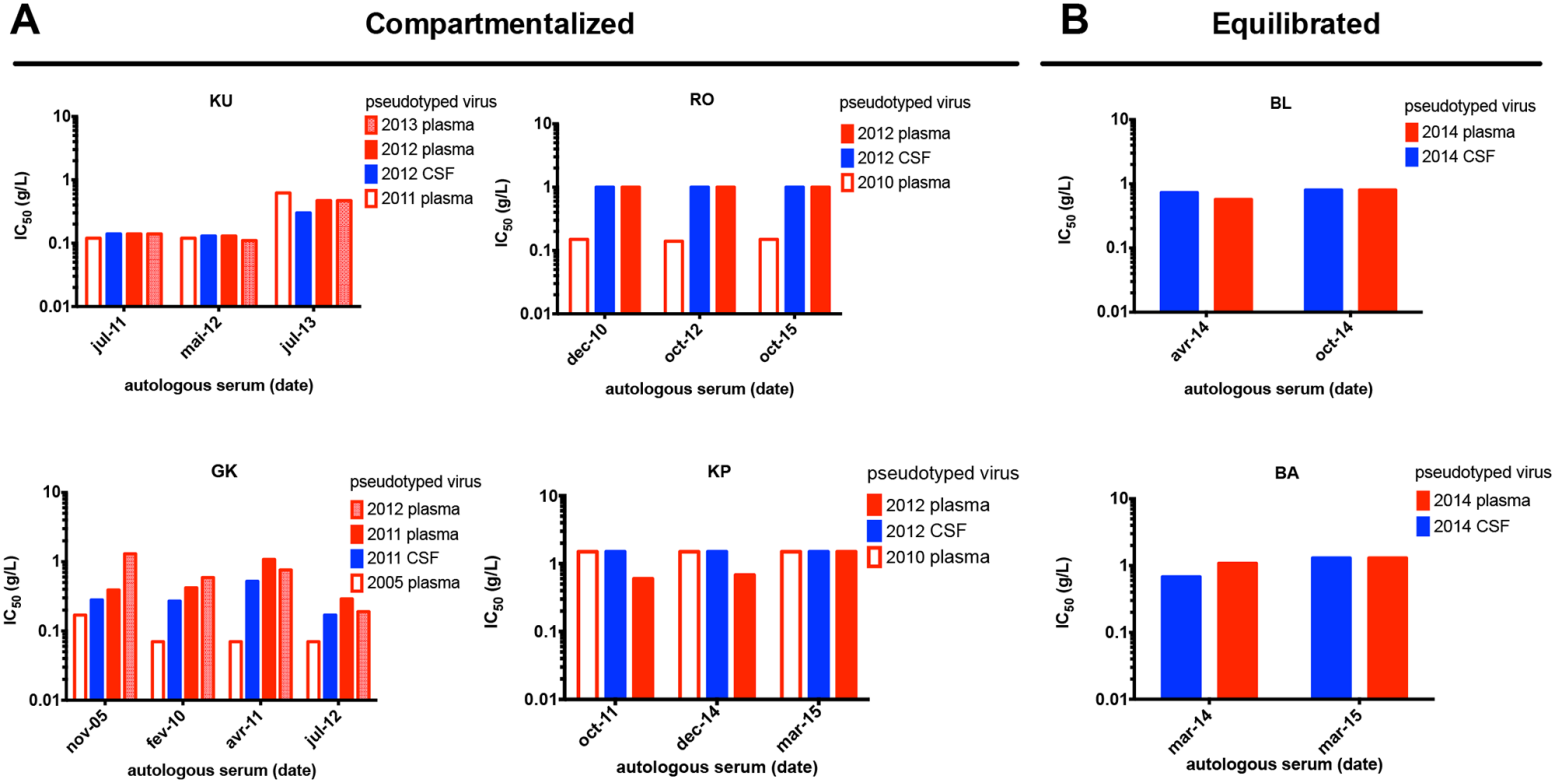
Sensitivity to neutralization by autologous antibodies of pseudotyped viruses from CSF and blood plasma samples from subjects with (A) or without (B) CSF compartmentalization. IgG were purified from sera taken at different time points for each patient (see abscissa). Pseudotyped viruses from contemporary CSF and plasma, as well as pseudotyped viruses from previous or subsequent plasma samples (see legend inserts), were exposed to serial dilutions of IgG purified from autologous sequential sera. The amount of IgG was normalized at the same starting concentration for all samples for each patient (approximately 1 g/L). Blue: CSF viruses. Red: plasma viruses.

Pseudotyped viruses expressing envelope glycoproteins representative of the quasi-species present in each sample were generated from the entire *env* gene amplified by RT-PCR from the plasma and the CSF of each patient. Infectious pseudotyped viruses were obtained for six of the nine subjects, including four individuals with longitudinal plasma samples and compartmentalized viral populations (KU, RO, GK, KP), and two subjects with equilibrated viral populations (BL, BA). We compared their sensitivity to autologous neutralization by measuring the 50% inhibitory concentration (IC_50_) by serially diluted purified IgG from autologous sera collected at different time points. As expected, the viral population sampled from blood plasma was either resistant (IC_50_ > 1 mg/mL) or moderately sensitive (IC_50_ = 0.13–1 mg/mL) to the contemporaneous autologous neutralization (Fig 4). In two cases (RO, GK), the blood plasma viral population at onset of neurologic symptoms was more resistant to autologous neutralization than the variants present in a blood plasma sample collected a few years before, illustrating the evolution to escape neutralization. Interestingly, the CSF viral population had a similar sensitivity to autologous neutralization than the contemporary blood plasma population for all the participants, regardless of the compartmentalization status (Fig 4). Despite the low concentration of antibodies in the CNS, the data suggest that the properties of the envelope glycoprotein of the neurotropic variants, even when compartmentalization occurs, do not result from more limited selective pressure by neutralizing antibodies in this compartment.

### The viral populations of the CSF display a broad range of sensitivity to neutralizing antibodies targeting the major antigenic sites

**Fig 5:**
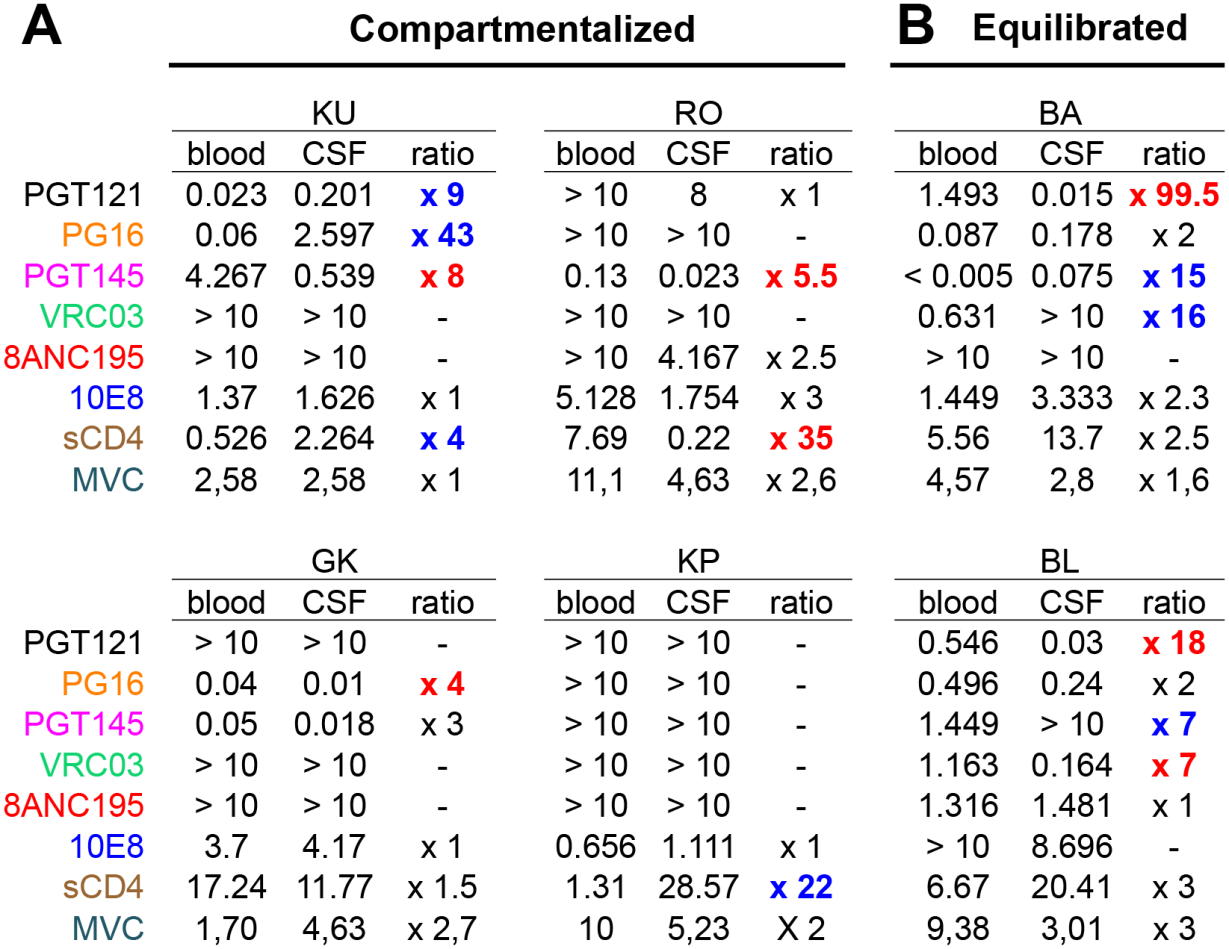
Sensitivity to broadly neutralizing antibodies and entry inhibitors of pseudotyped viruses from CSF and blood plasma samples from subjects with (A) or without (B) CSF compartmentalization. Paired pseudotyped viruses from CSF and blood were exposed to serial dilutions of bNAbs, sCD4 or maraviroc (MVC). Values indicate IC_**50**_ in μg/mL for bNAbs and sCD4 and in nmol/mL for MVC. Ratios showing a greater resistance of CSF viruses or a greater resistance of blood viruses (ratio > 3) are mentioned in blue or in red, respectively.

bNAbs target specific epitopes on the envelope spikes, thus interfering with the functionality of the envelope glycoproteins and preventing virus entry. We compared the sensitivity of the six pairs of contemporary plasma- and CSF-derived pseudotyped viruses to bNAbs representative of the major sites of vulnerability: PGT121, which targets a glycan-dependent epitope within or near the V3 loop of gp120; PGT145 and PG16, which recognize glycan-dependent quaternary epitopes in the V1V2 region; VRC03, which targets the CD4-binding site of gp120; 10E8 which targets the membrane-proximal external region (MPER) of gp41; and 8ANC195 which targets a conformational epitope overlapping both envelope subunits. The IC_50_ of each bNAb are recapitulated in Fig 5. Except for patient KP for whom the two viral populations were similarly resistant to five of the six bNAbs tested, the blood plasma- and CSF- populations of the other patients displayed a broad difference of sensitivities to neutralization, regardless of compartmentalization. No major difference was observed for 8ANC195 and 10E8. In contrast, major differences were observed for the bNAbs which target the three other sites of vulnerability. For instance, the IC_50_ of PG16 toward the CSF population of individual KU was 43 fold higher than toward the plasma population. Similarly, the IC_50_ of PGT121 toward the CSF population was highly reduced (99.5 fold increased sensitivity) and the IC_50_ of both PGT145 and VRC03 were increased by 16 and 15 fold respectively (i.e. increased resistance) when compared to the blood population in patient BA. The differences of sensitivity to bNAbs in the two sampled compartments were observed regardless of compartmentalization status, suggesting that the CSF populations had specific properties.

### The viral populations of the CSF display a broad range of sensitivity to sCD4 but not to maraviroc

We further investigated the phenotypic characteristics of the blood plasma- and CSF- viral populations by studying their sensitivity to sCD4 and maraviroc (MVC). All of them, either issued from plasma or CSF, were CCR5-tropic as determined by both genotypic and phenotypic assays. The sensitivity to MVC was almost similar in each pair of CSF- and blood- populations for all participants. The two cases with equilibrated viral populations presented sensitivity to sCD4 in the same range. However, two of four individuals with CSF-compartmentalized populations showed highly different sCD4 IC_50_ (Fig 5). The increased resistance to sCD4 was either for the blood population (RO) or the CSF population (KP).

### Compartmentalized CSF viral sequences share common features across patients and subtypes

**Fig 6:**
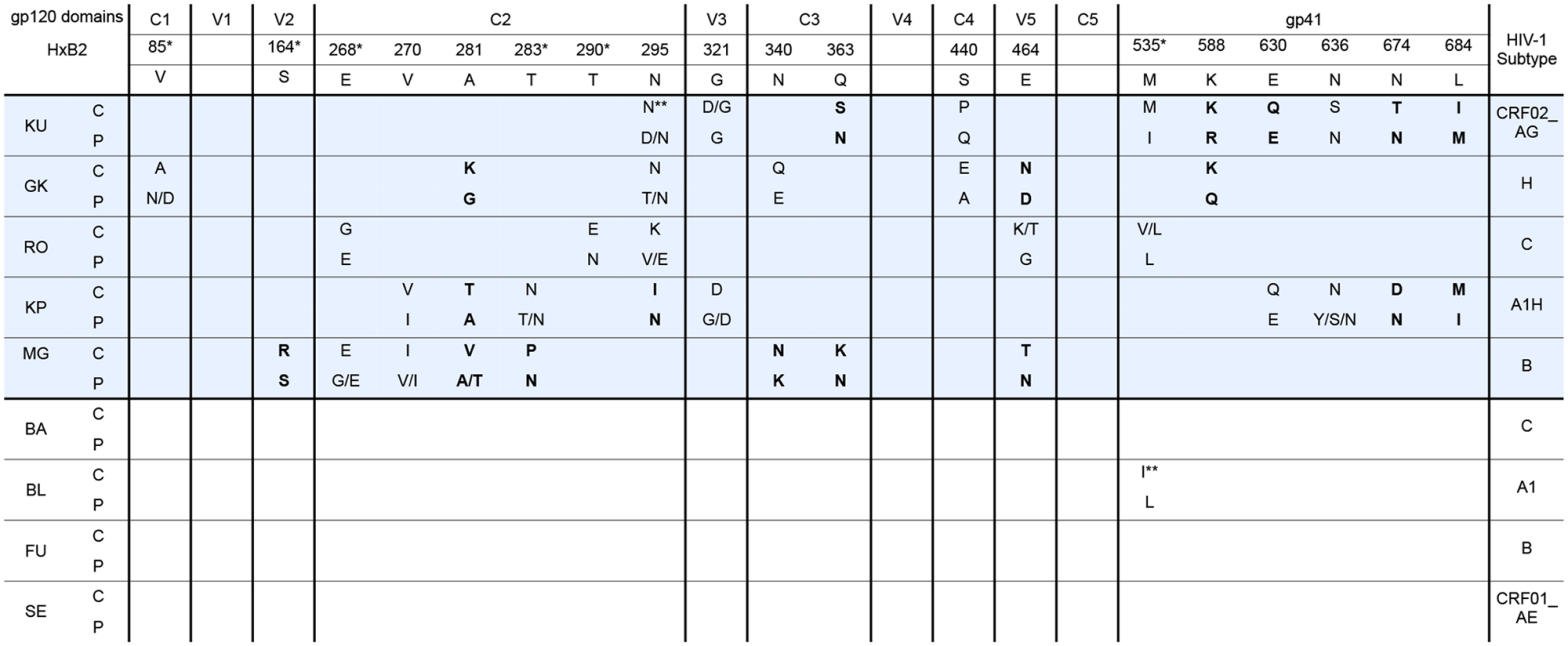
Sites identified as possibly involved in compartmentalization. Dominant amino acids (AA) at positions with statistically significant difference between CSF (C) and plasma (P) for at least 2 subjects or at position previously reported (*) are shown (*p* < 0.05). In bold, AA that met Bonferroni adjusted statistical significance. These positions were identified only in patients with compartmentalization (highlighted in blue). HxB2 gp160 numbering is indicated. (**) Differences observed at a position shared by several patients but below the significance threshold.

Using the *env* sequences from paired CSF and blood plasma samples, we tried to identify sequence signatures that would be specific of the localization to the CNS. After alignment completed by the GUIDANCE program to exclude positions for which there were uncertainties, we performed a signature pattern analysis using the Viral Epidemiology Signature Analysis (VESPA) software in order to examine the amino acid differences between CSF sequences and blood plasma sequences for each individual. A Fisher’s exact test was performed for each site to determine positions where statistically significant differences between the two compartments were observed and, for each protein alignment the number of variable sites was used to calculate the corrected Bonferroni p-value threshold. Using this approach, we identified several positions that either were shared by at least two individuals of our study or were found for one of our cases and reported in previous studies. There were thirteen positions in gp120 and six positions in gp41 where dominant amino acid in CSF sequences differed significantly from blood sequences (Fig 6). They were observed exclusively in the five patients with compartmentalization. In particular, six positions identified within C2 were similar or close to previously identified hot spots of CSF compartmentalization [15,21], suggesting their involvement in the replication of the virus in the CNS environment. Similarly, mutations at positions (or next to positions) 340 and 363 in C3 and 535 in gp41 were previously observed. The relevance of these hot spots is reinforced by the fact that the viral strains included in our study were related to highly divergent subtypes.

**Fig 7:**
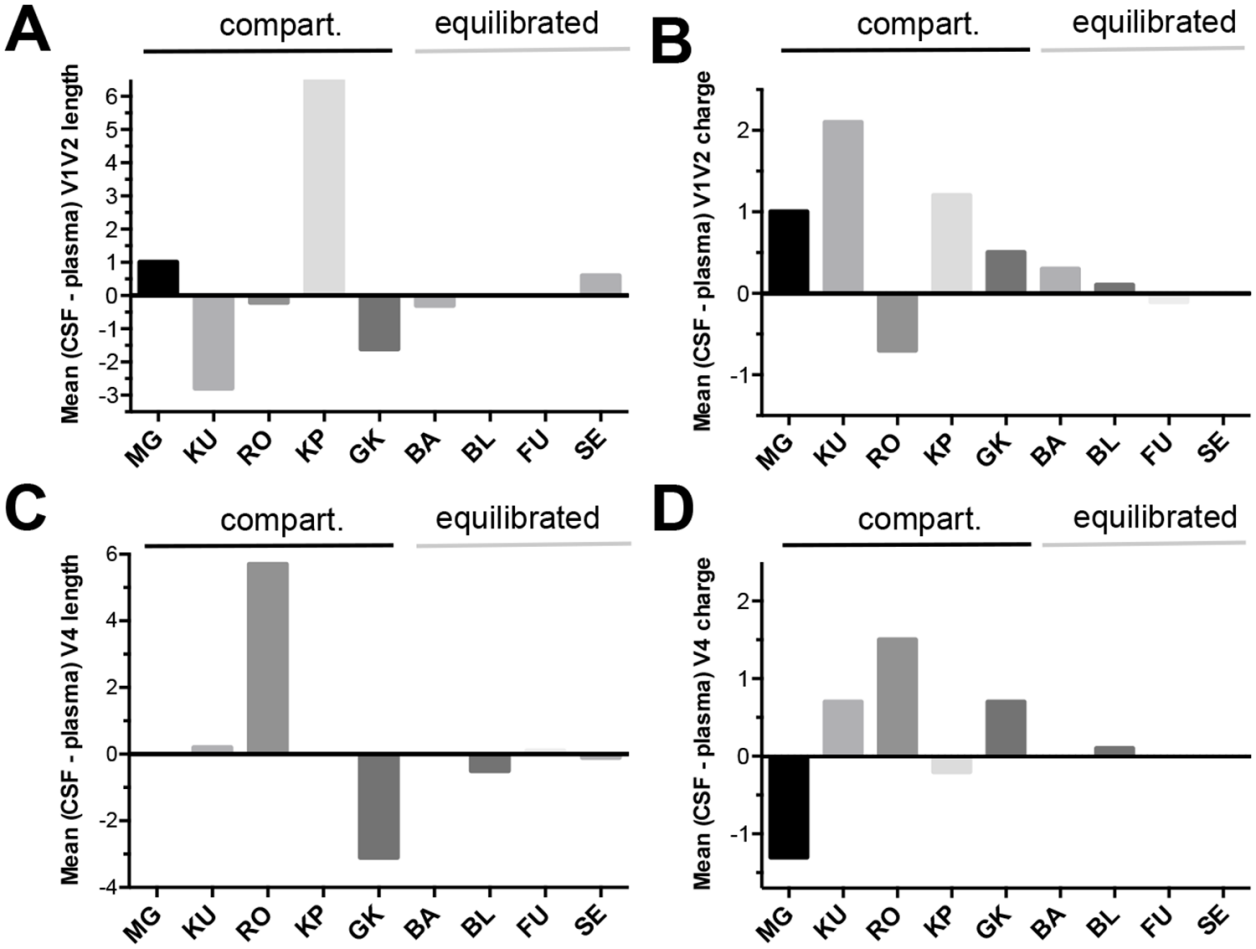
V1V2 and V4 loops length and charge differences of HIV-1 envelope glycoprotein in CSF compartmentalized viral populations. Histograms representing the difference between mean length or charge of V1V2 (A and B respectively) and V4 loops (C and D respectively) of single genome sequences from CSF compared to contemporaneous plasma. Patients are grouped according to evidence of compartmentalization in CSF (* = significant difference between CSF and plasma population by the Mann-Whitney test, *p* < 0.05).

However, usage of the GUIDANCE program could lead to an under-representation of relevant positions within the variable loops. Indeed, using the Variable Region Characteristics tool, major differences in both the length and the net charge of the V1V2 and the V4 loop between CSF and plasma sequences were observed for patients with compartmentalization whereas there were no or only minor differences for the patients with equilibrated viral populations (Fig 7). This suggests that these regions probably harbor sites that confer an advantage for replication in the CNS environment. In contrast there was no difference in length and net charges of the V3 and V5 regions (S2 Table). The number of PNGS within the variable regions did not differ between compartments and groups of patients (S3 Table).

## Discussion

The compartmentalization of HIV-1 in the CNS has been reported in association with severe neurocognitive stages as well as in milder forms of impairment [13,15–21]. Using phylogenetic analyses, compartmentalization has been observed in approximately half of chronically infected HIV-positive patients [14,19,21]. In addition, compartmentalization may occur early in the course of infection. Indeed, it was observed in 20% of adult cases already after four months of infection [20] and in 50% of children aged 18 to 36 months [19]. Using single genome sequencing (SGS) of full-length *env*, we found in the present work that 5 out of 9 adult patients suffering from various neurologic syndromes (55%) had evidence of viral compartmentalization in the CSF, in agreement with the previous studies.

The phenotypic correlates implicated in the replication in the CNS environment and the dynamics of this phenomenon in the course of the disease are far from understood. Some genetic characteristics of neuroadaptation across HIV envelope have been reported and shown to be related to an enhanced capacity to infect macrophages [19–20,22–24]. The Env glycoproteins are the main targets of the humoral response which drives their evolution toward resistance to NAbs. Therefore, one could suggest that, in contrast to the continuous escape observed in the blood, a lower selective pressure by antibodies in the CNS compartment would result in a viral population more sensitive to autologous neutralization compared to its blood counterpart, contributing to the compartmentalization. One of the main objectives of the present study was to answer this question. Our results show that the viral populations from the blood and from the CSF did not differ in their sensitivity to autologous neutralization for none of the patients, even for those with genetic compartmentalization. As expected due to the continuous escape largely described in the literature [26,36], most of the plasma viruses were resistant to the autologous sera taken at different time points, particularly to contemporaneous or previous serum samples. The fact that the CSF viruses were similarly resistant to neutralization compared to contemporaneous blood viruses suggests that the reduced concentration of antibodies in the CSF does not play a major role in driving the independent evolution of HIV-1 in the CNS, as it could be expected [14]. Our observation deserves to be analyzed considering compartmentalization not as a final fixed event but as a dynamic phenomenon, in the light of some previous findings. Indeed, bidirectional HIV migration between blood and CSF has been observed, suggesting a recirculation of viral populations from different anatomical sites over time [32,37,38]. Using SGS and Bayesian approaches, we investigated the temporal dynamics of compartmentalization in four patients with longitudinal plasma samples available. Our results show both the complexity and the diversity of this evolution between the two compartments, amplified still by the effect of ART. For two patients (RO and KP), the date of ART stop coincided with the diversification and the independent evolution of the CSF viral population. The diversity in CSF even reached a comparable level to that of the plasma viral population for patient RO. The TMRCA of CSF compartmentalized clusters were approximately 8 and 4 years for these patients, respectively, suggesting an independent replication on much longer periods of time than previously reported [20]. For one patient (GK) on the other hand, the CSF population had diverged from the plasma population since less than a year before, suggesting a recent seeding of the CNS. One limitation of our study is the unavailability of longitudinal CSF samples which would have provided the opportunity to analyze the evolution of the viral population within the CNS. In any case, these observations illustrate the viral migration from or to CSF over time and the dynamic replenishment of viral compartments, the autologous neutralization profile of the CSF viruses reflecting more their blood origin than an independent evolution sheltered from antibodies in the CNS compartment.

Highly potent human monoclonal bNAbs that have been isolated these recent years target epitopes known as sites of vulnerability on the Env spikes [36,39,40]. These sites are highly conserved within HIV-1 group M and therefore must play a major role for virus entry into the cell. Consequently, analyzing the sensitivity to bNAbs should provide indirect information on functional features of the Env glycoproteins. We used bNAbs binding each of the five following sites: the glycan-dependent V1V2 site, the glycan-dependent V3, the CD4 binding site, the membrane proximal external region of gp41 and the conformational site overlapping gp120 and gp41. We found major differences in neutralization sensitivity to at least one bNAb between the CSF viral populations and blood plasma viral populations for five of the six patients, the last case being resistant to all bNAbs. This suggests differences in Env glycoproteins functionality for CSF viral populations, even in the absence of compartmentalization. However, all the neutralization profiles of the virus pairs were different for each patient and we did not observe a common profile for CSF compartmentalized viruses nor for CSF viruses in general. Thus we could not identify any of the sites of vulnerability as playing a specific role in the evolution of CSF viruses. An unexpected consequence of our findings is the fact that HIV-1 variants present in the CSF can be more resistant to bNAbs than their blood counterpart. This must be emphasized in the light of recent clinical trials of immunotherapy during which escape variants were rapidly identified in patients receiving perfusions of bNAbs [41–44]. Our results warn us against the presence of variants resistant to bNAbs in reservoirs such as CNS which would be not detected in the blood. Considering the recirculation of the virus from/to the CSF and given the weak penetration of monoclonal antibodies through the blood-brain barrier, CNS appears as a perfect reservoir to fuel the escape from bNAbs-based therapy. Both our data and previous observations by others [41–44] emphasize that combinations of antibodies that target non-overlapping epitopes will be required for effective adjunct therapy.

Previous functional studies have shown that CNS/CSF-derived viruses may have adapted for efficient entry into brain macrophages which are known to express low levels of CD4 [19,20,24,45,46]. Although it was not in the main objectives of the study, we analyzed the sensitivity to MVC and sCD4 of the virus populations that we produced. All the viruses were R5-tropic and similarly sensitive to MVC, whatever their origin, CSF or blood plasma. In contrast, highly divergent sensitivities to sCD4 were observed between CSF viruses and their blood counterpart in a few cases, particularly for cases with CSF compartmentalization. However, we did not confirm recent findings from Arrildt et al. who found an increased sensitivity to sCD4 on a panel of seven macrophage-tropic clones isolated from CSF compared to their blood counterparts [46]. Taken altogether, our data concerning the sensitivity, either to bNAbs or to sCD4, illustrate both the differences of phenotypic properties of the CSF-derived viruses compared to their blood plasma counterpart and a broad diversity between each case, rendering improbable the existence of a single molecular support of the neurotropism within the Env glycoproteins.

Despite this diversity and complexity, we tried to identify some molecular features that would be associated with the neurotropism, particularly when compartmentalization was observed. As reported by others, we found that mean HIV-1 *env* diversity was statistically lower in CSF- than in plasma-derived variants in some patients with compartmentalized virus [21]. Focusing on the different Env regions we found that the mean diversity between CSF- and plasma- variants was significantly increased in V1V2, C2 and gp41 regions for patients with compartmentalization, suggesting that residues localized within these regions might be involved in neurotropism. The possible involvement of the V1V2 loop was reinforced by the differences in both its length and its net charge between CSF and plasma sequences for patients with compartmentalization. We tried to identify more precisely sites and/or sequence signatures that would be specific of the localization to the CNS. For each individual, signature pattern analysis was performed using the VESPA software that examined the amino acid differences between CSF sequences and blood plasma sequences. We identified thirteen positions in gp120 and six positions in gp41 where dominant amino acid in CSF sequences significantly differed from the blood sequences and were shared by at least two patients. Numerous positions clustered in the C2 region, some of them having already been identified by others such as positions 268, 281, 283 [15,21]. The compartmentalization at position 281 is noteworthy as it was shared by three viruses of different subtypes (B, H, A1H). This position was shown to be in direct contact with CD4 [47,48] suggesting that it could play an important role in HIV-1 adaptation to low-CD4 tropism. Position 283, close to 281 and also in direct contact with CD4, was already shown as playing a key role in modulating CD4 dependence, macrophage tropism, brain infection and dementia [15]. Interestingly, position 268 which was identified for two patients in our study and two other patients in Evering’s study [21], was also identified in resistant variants that emerged after treatment with VRC01, a bNAb targeting the CD4 binding site of gp120 [42]. Several patients had compartmentalized position in the loop V5 (position 462 to 465, Fig 6), a region in direct contact with CD4 and where most of the resistance mutation to VRC01 occurred [41]. We identified other positions, particularly in C3 and gp41, which were also found in other studies, suggesting their belonging to hot spots for CNS compartmentalization. The fact that the viral strains included in our study were related to highly divergent subtypes, in contrast to previous studies that included patients mainly infected by subtype B strains, reinforces the relevance of the sites identified.

In this study, we analyzed both the genotypic properties and the phenotypic properties of paired CSF and blood plasma samples from patients with HIV-1 associated neurologic syndromes. Most of the published previous studies analyzed the phenotypic properties of the Env glycoproteins of CNS/CSF variants using *env* clones isolated from brain or CSF. Instead of working with clones, which allow to identify the individual phenotypes of the envelope variants that are present in each compartment, we have chosen to perform our study with pseudotyped viruses expressing envelope glycoproteins representative of the viral quasi-species present in each compartment. Although we cannot exclude that bulk PCR can amplify certain predominant variants selectively and generate recombination artefacts, we believe that this approach allows to be more representative of the physiological situation occurring in each patient.

In conclusion, our study provides additional proofs of the complexity of the compartmentalization of HIV-1 and suggests that different independent factors are involved in HIV-1 infection of the CNS. Among them, the viral migration from or to CNS over time, eventually modified by ART treatment or its interruption, and the interaction of the HIV-1 envelope glycoproteins with CD4 would deserve to be analyzed in depth. We also showed that selective pressure by autologous NAb does not appear as a major driver of the HIV evolution in the CNS, and that CNS could be a reservoir of bNAb resistant viruses, an observation that should be kept in mind when considering passive immunization as a potential strategy to the therapy and cure of HIV-1 infection.

## Materials and methods

### Study participants and clinical specimens

HIV-1 infected patients were selected based on the availability of contemporary CSF and plasma samples with viral load > 1 000 copies/mL. They were hospitalized for various neurologic disorders for which a lumbar puncture was required for diagnostic purpose. Diagnoses and CSF characteristics for the nine individuals are detailed in S1 Table. No common opportunistic pathogen was identified for these individuals except patient SE who suffered from cerebral toxoplasmosis. For the present study, samples were analyzed retrospectively after all biological testing for clinical management had been performed. We were able to include nine patients for which paired CSF and plasma samples met the criteria. Longitudinal plasma samples were available for four patients, but no additional sequential CSF sample was available for any of them. All were chronically infected and rather deeply immunosuppressed at time of the lumbar puncture (median CD4+ T-lymphocyte count of 179 cells/mm^3^; range 23–319). They were not on antiretroviral treatment (ART) or, if treated, had a poor adherence to treatment.

### Ethics statement

Patients were assigned identification codes (2 letters) that were unique to this analysis and could only be linked back to the original data by a single authorized personnel. The database was approved by the French watchdog committee (#2017–056). Written informed consent was collected from patients who were still in care in our hospital. The Institutional Board of the University Hospital of Tours (Espace de Réflexion Ethique Région Centre) reviewed the study for approval (#2017–021).

### Single genome amplification and sequencing

HIV-1 RNA was extracted from plasma and CSF (150 μL) using the Nucleospin RNA Virus kit (Macherey-Nagel). Samples with viral loads <10,000 copies/ml were first pelleted by ultracentrifugation to increase the viral RNA recovery. Reverse transcription (RT) was carried out using 16 μL of viral RNA, outer primer ExtM3 (5’- CTTRTAAGTCATTGGTCTTAAAGGYAG-3’) and the Superscript III First strand synthesis system (Invitrogen) according to manufacturer’s instructions. Single genome amplification (SGA) of the full-length HIV-1 *env* was conducted as described elsewhere [49,50]. Briefly, nested PCR were carried out using endpoint dilutions of the cDNA amplified with Platinum Taq High Fidelity polymerase (Invitrogen), using the outer primers pair ExtM5 (5’-ATGGCAGGAAGAAGCGG-ARRC-3’) and ExtM3 and the inner primers pair IntM5XE (5’-AATTCTCGAGAATTCAGAAAGAGC-AGAAGACAGTGGCAATG-3’) and IntM3MX (5’-GGCCACGCGTCTAGACTACTTTTTGACCACT-TGCCMCCCAT-3’) with the following conditions: 2 min at 94°C, then 35 cycles of 15 s at 94°C, 30 s at 55°C and 3 min at 68°C, and a final extension step of 10 min at 68°C. HIV-1 *env* gene products were directly sequenced according to the Dye Terminator cycle sequencing protocol (Applied Biosystems, Foster City, Calif.) Each chromatogram was carefully inspected. Sequences with double peaks, sequences with frameshift mutations or premature stop codons were excluded from analysis. All sequences have been submitted to GenBank and assigned accession numbers KY825261 to KY825713.

### Phylogenetic analyses

Multiple sequence alignments of the HIV *env* region were built with Clustal-W [51] and then manually edited using BioEdit [52]. Phylogenetic trees were constructed on MEGA6 [53] using the neighbor-joining method.

For each individual, compartmentalization between blood and CSF viral populations was evaluated using a panel of methods available on HyPhy software package [54]: the Wright measure of population subdivision (F_st_) [34], the nearest-neighbor statistic (Snn) [35], the Slatkin-Maddison test [33]. Compartmentalization was affirmed when all tests yielded significance (*P* < 0.01) [31,32].

### Bayesian analyses

A Bayesian Markov chain Monte Carlo (BMCMC) approach was used to reconstruct phylogenies when longitudinal plasma samples were available. Sampling dates were used to calibrate the time scale and the site of sampling was implemented. Briefly, BMCMC chains of 50 million generations were performed for each analysis with a GTR + Γ4 substitution model under an extended Bayesian skyline plot coalescent model, logging every 1000 and with a 10% burn-in. All analyses were performed using an uncorrelated lognormal relaxed molecular clock implemented in the BEAST software package [55]. Tracer (version 1.5) was used to check for convergence and trees were visualized with FigTree (version 1.4.0).

### Sequence analyses

The mean average pairwise distance (APD) within each compartment and the mean average pairwise distance between compartments (BC-APD) were determined using the distance matrix in MEGA6 from each multiple amino acid sequence alignment, with a JTT plus Gamma model. Signature pattern analysis was performed using the Viral Epidemiology Signature Analysis (VESPA) programme on the Los Alamos HIV database site [56]. For each individual, the amino acid alignment of CSF-derived SGS (input) was compared to the amino acid alignment of contemporaneous plasma-derived SGS (background). The reliability of multiple sequence alignments was evaluated using the web-based GUIDANCE program (http://guidance.tau.ac.il/overview.html) [57] For each protein alignment the number of variable sites was used to calculate the corrected Bonferroni p-value threshold [21]. The Variable Region Characteristics tool was used to reports features of the gp120 variable loops. Potential N-linked glycosylation sites (PNGS) were identified using the N-Glycosite web server [58]. Both programs are available on the Los Alamos HIV database site (https://www.hiv.lanl.gov/).

### Production of env-pseudotyped viruses

Full length (gp160) *env* genes were amplified by nested RT-PCR as previously reported, using the same primers as described above for SGA. Reverse transcription (RT) was carried out using outer primer ExtM3 and the Superscript III First strand synthesis system (Invitrogen). It was followed by the first round of PCR, using the Platinum PCR SuperMix High Fidelity (Invitrogen) with the following conditions: 2 min at 94°C, then 35 cycles of 15 s at 94°C, 30 s at 55°C and 3 min at 68°C, and a final extension step of 10 min at 68°C. A 5 μL aliquot of the products from the first round of PCR was then used as template for the second round of amplification under the same cycling conditions. Each fragment was approximately 2.6 kb in length, spanning the entire open reading frame of the HIV-1 gp160 polyprotein. The inner primers IntM5XE containing XhoI and EcoRI sites and IntM3MX containing MluI and XbaI sites [59], the amplification products were digested with XhoI and XbaI or XhoI and MluI restriction enzymes (New England Biolabs), then purified by agarose gel electrophoresis and ligated into the XhoI and XbaI or XhoI and MluI digested pCI expression vector (Promega). The resulting pCI-env plasmids representing the amplified virus populations were propagated by transformation of Electromax DH5a electrocompetent *Escherichia coli* (Invitrogen). Library of pCI-env plasmids were purified from transformed cultures using silica column chromatography (Macherey-Nagel). Env-pseudotyped viruses were produced as previously described [59] by cotransfecting 5.10^6^ 293T cells with 4 μg of each patient-derived pCI-env library and 8 μg of pNL4.3.LUC.R_E_ [60] using FuGene-6 transfection reagent (Promega). Virus stocks were harvested 72 h later, purified by filtration (0.45 mm filter) and stored as aliquots at -80°C. Viral infectivity was monitored by infection of 1.10^4^ TZM-bl cells, with serial five-fold dilutions of viral supernatants in quadruplicate, in the presence of 30 mg/mL DEAE-dextran. Infection levels were determined after 48 h by measuring the luciferase activity of cell lysates using the Bright-Glo luciferase assay (Promega) and a Centro LB 960 luminometer (Berthold Technologies) and the TCID50 was calculated as described previously [61].

### IgG purification

IgGs were purified from 1 to 2 mL of plasma samples by gravity-flow protein G affinity chromatography (GE Healthcare). The elution fraction was then desalted and concentrated *via* multiple round centrifugations with Amicon Ultra centrifugal filter devices (Millipore) in normal saline solution. IgG concentration was measured with BN ProSpec analyzer (Siemens).

### Neutralization assay and sensitivity to sCD4

Sensitivity to autologous IgGs, to bNAbs and to soluble CD4 (sCD4; Progenics Pharmaceuticals) of the pseudotyped viruses was assessed as previously described [62]. After titration, virus stocks were diluted to 400 TCID50/mL. Aliquots of 50 μL were then incubated for 1 h at 37°C with 100 μL of 3-fold serial dilutions of either purified IgG normalized across patient’s sera (starting 1.3 μg/mL for GK, 0.8 μg/mL for KU, 1 μg/mL for RO, 1.5 μg/mL for KP, 1.3 μg/mL for BA and 0.8 μg/mL for BL), or bNAbs (PGT121, PGT145, PG16, VRC03, 10E8, 8ANC195, starting at 10 μg/ml; IAVI and NIH AIDS Reagent Program) or sCD4 (starting at 10 μg/mL; NIH AIDS Reagent Program). The virus-antibody mixture was then used to infect 10,000 TZM-bl cells in the presence of 30 μg/ml DEAE-dextran. Infection levels were determined after 48 h by measuring the luciferase activities of cell lysates. Results were expressed as the mean values of the assays performed in duplicates. IC50 values were defined as the reciprocal of the serum dilution or the antibody concentration required to reduce RLUs by 50%.

### Determination of coreceptor usage and sensitivity to maraviroc

Prediction of the coreceptor usage was performed through Geno2pheno algorithm for each sequence [63]. A false positive rate below 10% was predictive of X4 tropism. Coreceptor usage was phenotypically assessed by measuring the ability of the pseudotyped viruses to infect U373.R5 and U373.X4, expressing exclusively either CCR5 or CXCR4 coreceptor respectively [64]. Cells were plated at 1.5 x 10^4^ cells per well one day prior to infection with viruses diluted to 400 TCID50/mL. Successful infection led to production of luciferase activity in target cells, measured 48 hours post inoculation. Pseudotyped viruses with AD8 (pAD8) or NL4.3 (pNL4.3) envelope were used respectively as R5 tropism and X4 tropism positive controls. A pseudotyped virus obtained by co-transfection of empty-pCI expression vector was used as negative control. Technical cutoff for R5 and X4 coreceptor usage capacity was calculated as the means plus two standard deviations (SD) of RLU detected after U373.R5 infection by pNL4.3 and U373.X4 infection by pAD8 respectively.

For MVC inhibition, 8.10^3^ TZM-bl cells per well were prepared the day prior infection. Cells were first treated for 1h at 37°C with 150 μL of three-fold serial dilutions of MVC (6 μM to 0.3 nM) before adding 50 μL of pseudotyped viruses normalized to 400 TCID50/mL. 100 μl of DMEM medium supplemented with 30 μg/mL DEAE-dextran were then added to cells. IC50 values were defined as the reciprocal of the MVC concentration required to reduce RLUs by 50%.

## Supporting information

S1 Fig Maximum Likelihood tree representing HIV-1 *env* sequences from nine subjects suffering from neurological disease. Sequences from both compartments (plasma and CSF) and all time points were aligned with reference sequences from HIV-1 major subtypes. Each subject formed a distinct cluster, showing the absence of contamination. HIV-1 quasispecies infecting each patient belonged to a variety of clades. Bootstrap analysis (500 replications) was used to test the reliability of the branching order. Bootstrap values ≥ 0.70 are represented by an asterisk. CPZ.CM.CAM5 was used as an outgroup.
(TIF)

S1 Table CNS diagnoses and CSF analyses for the nine patients. (TIF)

S2 Table Characteristics of HIV-1 env variable regions. For each paired CSF/blood plasma single genome sequences dataset, mean length, number of potential N-glycosylation sites and charge of V1V2, V3 and V4 variable regions are indicated, as well as the mean difference between compartments.
(TIF)

S3 Table Total number of potential N-glycosylation sites. For each paired CSF/blood plasma single genome sequences dataset, mean number of potential N-glycosylation sites on HIV-1 Env is indicated, as well as the mean difference between compartments.
(TIF)
