# Supplementary figures and images for: Probing the compartmentalization of HIV-1 in the central nervous system through its neutralization properties
Source: PLoS One. 2017 Aug 25;12(8):e0181680. doi: 10.1371/journal.pone.0181680 (PMC5571919; doi:10.1371/journal.pone.0181680)

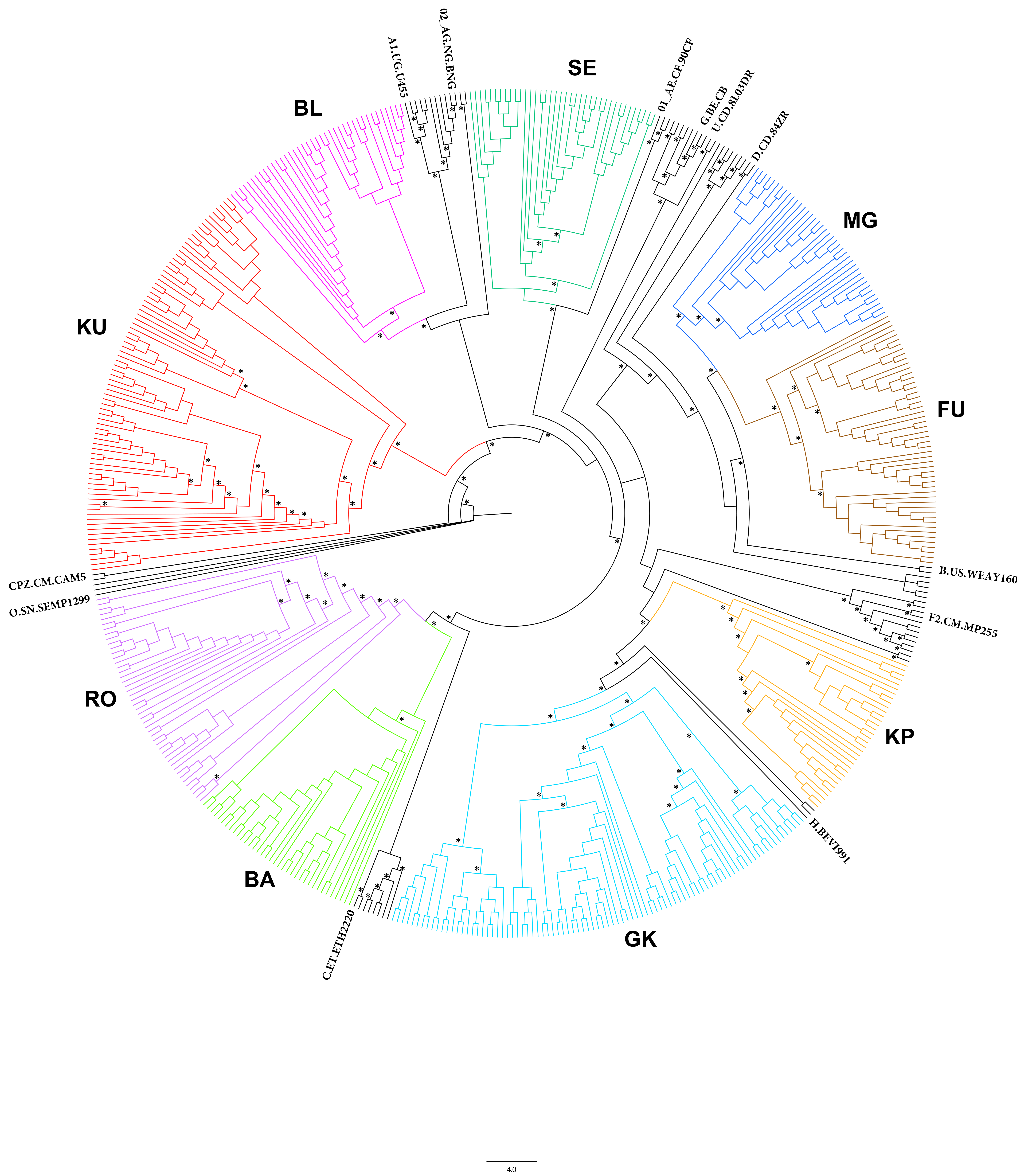

Supplement: S1 Fig — Sequences from both compartments (plasma and CSF) and all time points were aligned with reference sequences from HIV-1 major subtypes. Each subject formed a distinct cluster, showing the absence of contamination. HIV-1 quasispecies infecting each patient belonged to a variety of clades. Bootstrap analysis (500 replications) was used to test the reliability of the branching order. Bootstrap values ≥ 0.70 are represented by an asterisk. CPZ.CM.CAM5 was used as an outgroup. (TIF) [file pone.0181680.s001.tif]

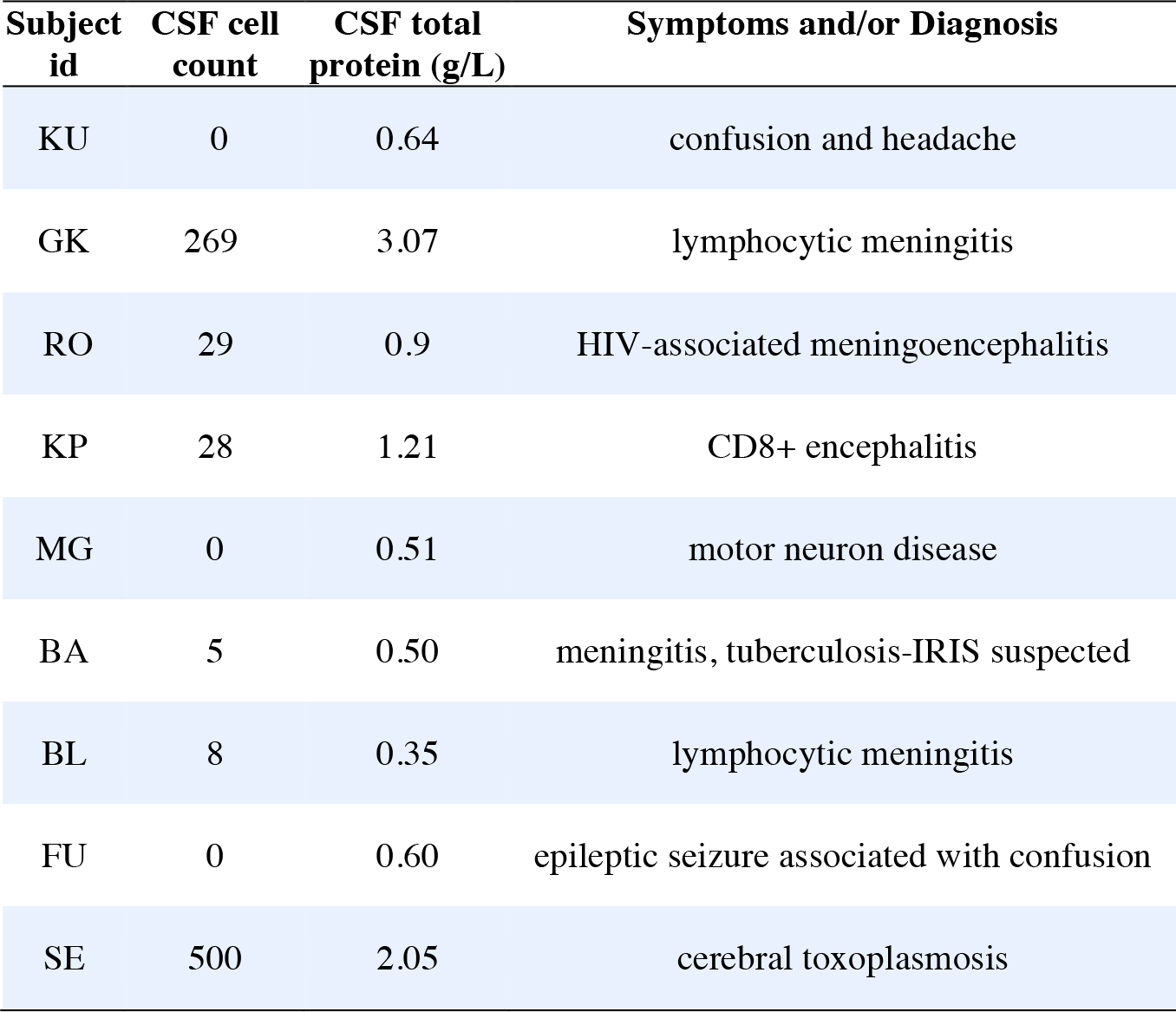

Supplement: S1 Table — (TIF) [file pone.0181680.s002.tif]

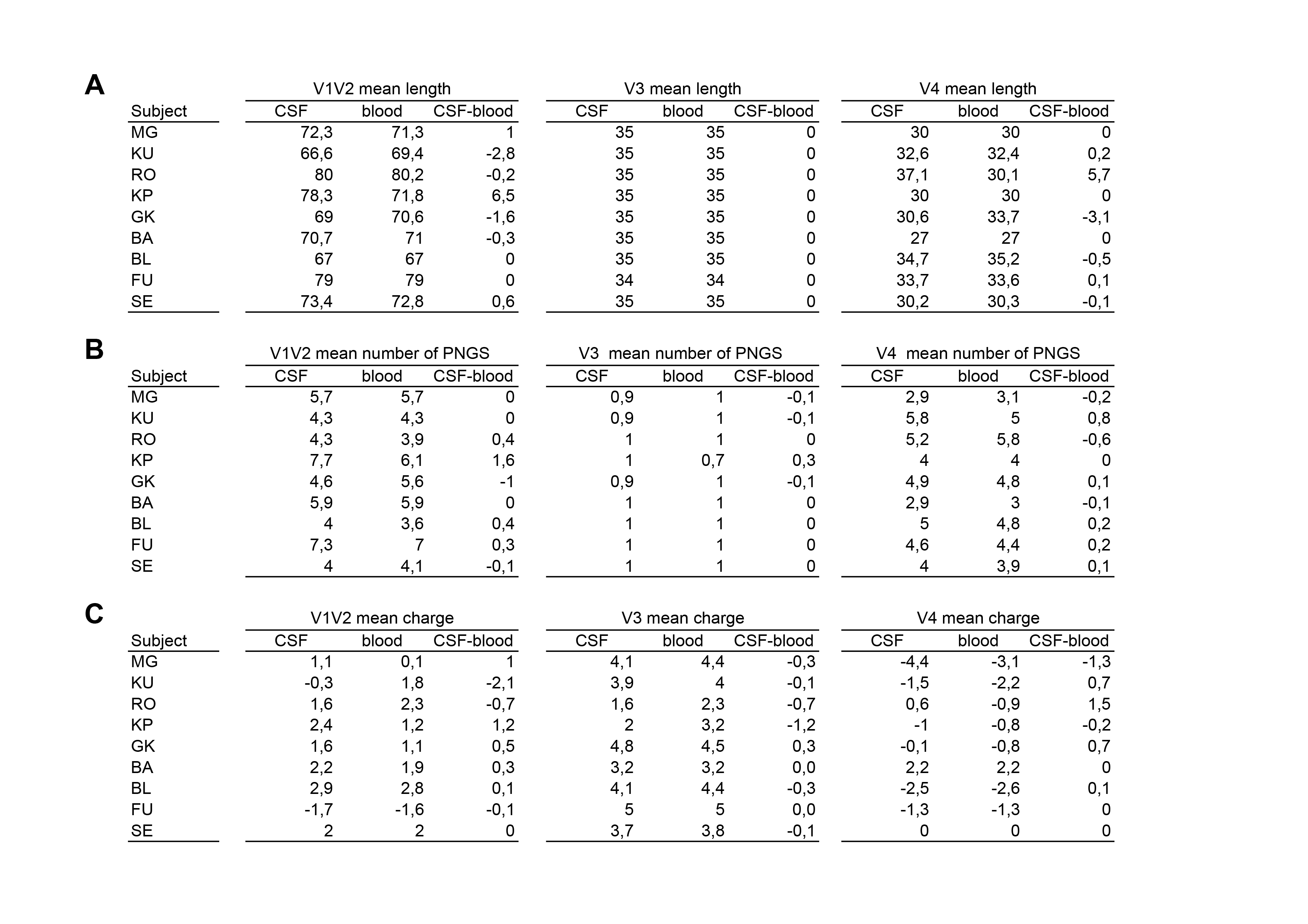

Supplement: S2 Table — For each paired CSF/blood plasma single genome sequences dataset, mean length, number of potential N-glycosylation sites and charge of V1V2, V3 and V4 variable regions are indicated, as well as the mean difference between compartments. (TIF) [file pone.0181680.s003.tif]

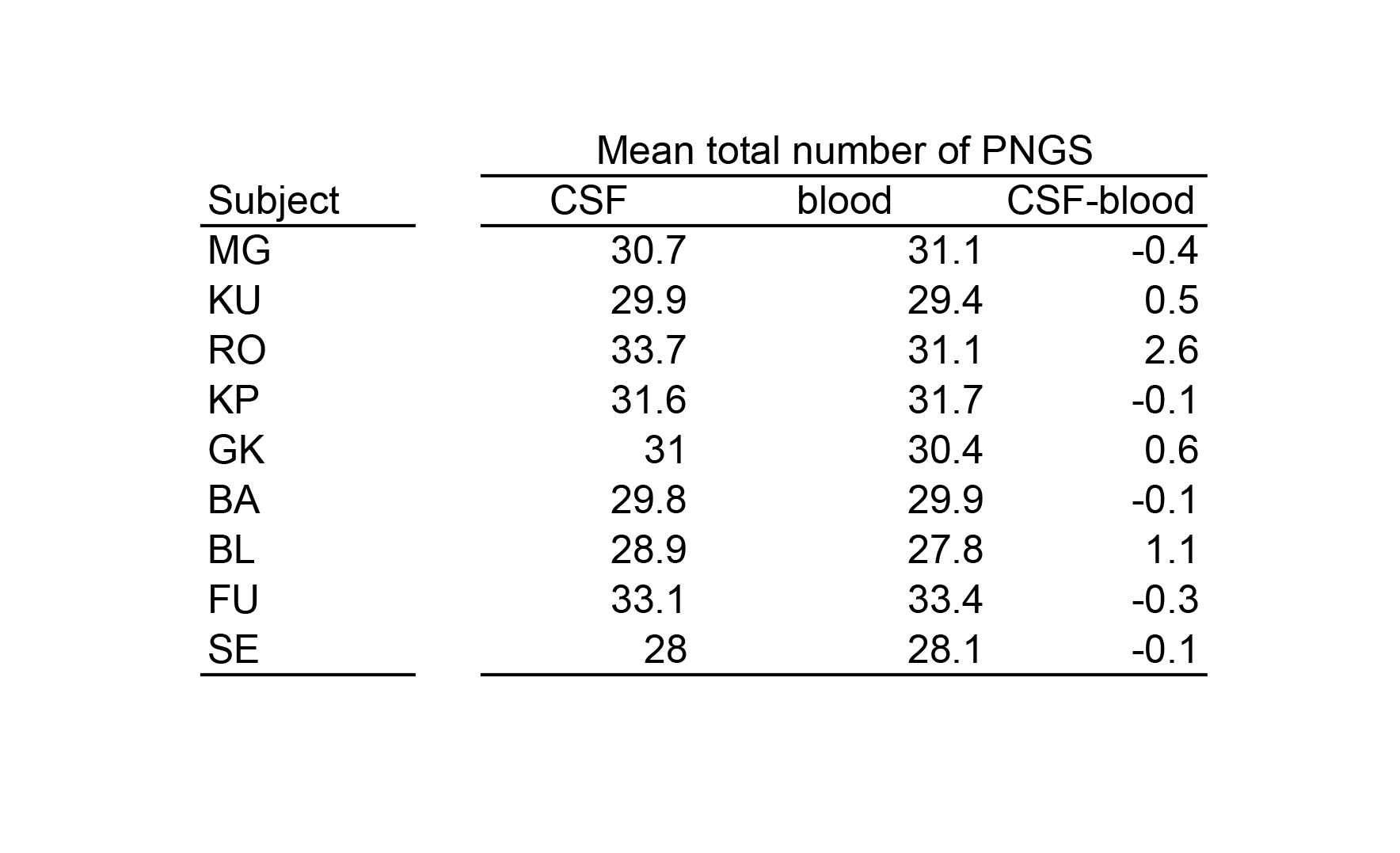

Supplement: S3 Table — For each paired CSF/blood plasma single genome sequences dataset, mean number of potential N-glycosylation sites on HIV-1 Env is indicated, as well as the mean difference between compartments. (TIF) [file pone.0181680.s004.tif]
